# Attenuated β-adrenergic response in calcium/calmodulin-dependent protein kinase IV-knockout mice

**DOI:** 10.1371/journal.pone.0249932

**Published:** 2021-04-15

**Authors:** Manabu Murakami, Agnieszka M. Murakami, Yasushi Matsuzaki, Daisuke Sawamura, Takayoshi Ohba, Ichirou Miyoshi, Shirou Itagaki, Hiroyuki Sakagami

**Affiliations:** 1 Department of Pharmacology, Graduate School of Medicine, Hirosaki University, Hirosaki, Japan; 2 Department of Dermatology, Graduate School of Medicine, Hirosaki University, Hirosaki, Japan; 3 Department of Cell Physiology, Akita University School of Medicine, Akita, Japan; 4 Department of Animal Care, Tohoku University School of Medicine, Aoba-Ku, Sendai, Japan; 5 Collaboration Center for Community and Industry, Sapporo Medical University, Sapporo, Japan; 6 Department of Anatomy, Kitasato University School of Medicine, Sagamihara, Japan; University of Minnesota, UNITED STATES

## Abstract

In the present study, we examined the importance of Ca^2+^/calmodulin-dependent protein kinase IV (CaMKIV) in the regulation of cardiac function using genetically modified CaMKIV-null mice. RT-PCR analysis revealed decreased expression of voltage-dependent calcium channels in the cardiac myocytes of CaMKIV-null mice compared with wild-type mice. CaMKIV-null mice showed shortened QT time on electrocardiograms. Pharmacological analysis revealed decreased responsiveness to the β-adrenergic blocker propranolol in CaMKIV-null mice, whereas the plasma norepinephrine level was not affected. CaMKIV-null mice showed decreased baroreflex on electrocardiograms. Heart rate variability analysis showed unstable R-R intervals, a decreased low frequency power/high frequency power (LF/HF) ratio, and increased standard deviation of the normal to normal R-R intervals (SDNN) in CaMKIV-null mice, suggesting decreased responsiveness to β-adrenergic stimulation in CaMKIV-null mice. Atrial contraction analysis and cardiac action potential recording showed a decreased response to the β-adrenoceptor agonist isoproterenol in CaMKIV-null mice. Furthermore, fluorescence imaging in a CRE-hrGFP assay revealed a decreased response to isoproterenol in CaMKIV-null cardiac myocytes. Taken together, our data strongly suggest a significant effect of CaMKIV gene ablation on cardiac β-adrenergic signal transduction.

## Introduction

Calcium (Ca^2+^)/calmodulin-dependent protein kinase IV (CaMKIV) is a multifunctional, serine/threonine protein kinase that is activated by intracellular Ca^2+^. CaMKIV phosphorylates cAMP response element binding protein (CREB), and this phosphorylation is terminated by calcineurin [1]. The distribution of CaMKIV may be limited to the brain, T lymphocytes, and postmeiotic germ cells, as well as cardiac myocytes [2–6]. Among the different Ca^2+^-dependent protein kinases, CaMKIV is detected predominantly in the nuclei of neurons [7] and may therefore play a unique role in CREB phosphorylation and in regulation of the expression of various genes. CaMKIV is a component of a major CREB pathway [8]. It has been hypothesized that CaMKIV plays a significant role in the nervous system. Because CaMKIV is localized with CREB in the nucleus, it appears to be important for CREB-mediated transcriptional activation. Due to its high expression in cardiac myocytes, CaMKII is thought to be the main CaMK in cardiac myocytes, but the importance of CaMKIV in these cells remains obscure.

The sympathetic nervous system and its neurotransmitter norepinephrine are associated with the “fight or flight” response. Sympathetic facilitation of cardiac function is mediated by norepinephrine-induced stimulation of β-adrenoceptors. The molecular mechanism underlying β-adrenergic stimulation depends on the modulation of Ca^2+^ channel currents by the G-protein-coupled pathway [9]. As calcium/calmodulin kinase participates in CREB phosphorylation, it is possible that CaMK might affect CREB-related pathways.

Increased intracellular calcium concentration is considered an important factor in cardiac hypertrophy. Calcium agonists, Ca^2+^ ionophores, and extracellular Ca^2+^ cause hypertrophic changes in primary cultures of cardiac myocytes [10–12]. There is substantial evidence that the intracellular Ca^2+^-binding protein, calmodulin, is a key regulator of cardiac hypertrophy. Overexpression of calmodulin in the hearts of transgenic mice induces cardiac hypertrophy [13]. Treating cultured cardiomyocytes with the calmodulin antagonist W-7 prevents hypertrophy in response to α-adrenergic stimulation and Ca^2+^ channel agonists [10]. Cardiac CaMK activity was also reported to be elevated in patients with dilated cardiomyopathy [14, 15]. However, whether CaMKIV signaling is involved in hypertrophic growth *in vivo* has not been investigated.

To date, three independent strains of mice with a null mutation in CaMKIV [16, 17] and transgenic mice bearing the dominant negative form of the CaMKIV gene have been generated [2]; however, the involvement of CaMKIV in the cardiovascular system has not been analyzed in these animal models. The present study was performed to examine the importance of CaMKIV in the cardiovascular system using CaMKIV-null mice. Here, we examined the cardiovascular system and sympathetic nerve control to determine the effects of CaMKIV deficiency.

## Materials and methods

### CaMKIV-deficient mice

A CaMKIV-null mouse line was maintained in the hemizygous state [17]. The animals were housed under a constant 12-h light/dark cycle with free access to food and water throughout the study. All experimental procedures were approved by the Institutional Animal Care and Research Advisory Committee of the Hirosaki University School of Medicine. To isolate hearts, the mice were anesthetized with an intraperitoneal injection of a mixture of medetomidine hydrochloride (0.315 mg/kg), midazolam (2.0 mg/kg), and butorphanol tartrate (2.5 mg/kg). The mice were first anesthetized by inhaling 80% carbon dioxide and 20% oxygen prior to the intraperitoneal injection to prevent any pain associated with the injections.

### Reverse Transcription–Polymerase Chain Reaction (RT-PCR)

Poly(A)+ RNA was isolated from cells using TRIzol reagent (Invitrogen, Carlsbad, CA, USA) and Oligotex-dT30 (Takara, Shiga, Japan). Reverse transcription was performed using a first-strand cDNA synthesis kit (SuperScript II Reverse Transcriptase; Invitrogen). PCR amplification was performed using GoTaq Green Master Mix (Promega, Madison, WI, USA).

Sequences of CaMKIV, CaMKII, CREB, adrenergic β1 receptor (β1), muscarinic type-2 receptor (M2), α-myosin heavy chain (α-MHC), β-myosin heavy chain (β-MHC), myocyte enhancer factor-2 (MEF2), GATA binding protein 4 (GATA4), brain natriuretic peptide (BNP), atrial natriuretic factor (ANF), and CaV1.2 (voltage-dependent calcium channel [VDCC]) were amplified (34 cycles) using specific primer pairs (S1 Table). All RT-PCR reactions were analyzed independently at least four times.

### Isolation of cardiomyocytes and whole-cell immunostaining

Adult atrial and ventricular cardiomyocytes were dissociated enzymatically. Atria and ventricles from three hearts were dissected under a microscope, minced using fine scissors, and transferred to isolation solution containing collagenase I (1.96 mg/mL) and trypsin I (0.78 mg/mL). The myocytes were isolated at least twice from each mouse line.

Whole-cell immunofluorescence labeling of isolated cardiac myocytes was performed using an anti-CaV1.2 antibody at a dilution of 1:100 (Alomone Labs, Jerusalem, Israel) as the primary antibody; visualization was performed using fluorescein-conjugated polyclonal goat anti-guinea pig IgG at a dilution of 1:200.

### Histological examination

Heart tissues were fixed in 4% (*v/v*) paraformaldehyde in 0.01 M sodium phosphate buffer (pH 7.2) overnight at 4°C, embedded in paraffin wax, and cut into 3 μm thick sections. For histological analysis, these sections were stained with hematoxylin and eosin and observed under an optical microscope.

Cross-sectional area (CSA) measurements were obtained from a minimum of 50 cardiac myocytes from wild-type and CaMKIV-null mice. Four images from non-overlapping regions of each CSA of tissue stained with hematoxylin and eosin were used for the CSA measurements. The mean fiber CSA of the respective fiber types was determined by planimetry.

### Western blot analysis

Partially purified membranes were prepared in 50 mM Tris-HCl buffer (pH 7.4) containing a mixture of protease inhibitors for the CaV1.2 expression analysis. Aliquots (100 μg) of the homogenate from each mouse were resolved by 7.5% sodium dodecyl sulfate-polyacrylamide gel electrophoresis and subjected to western blotting. Commercially available polyclonal antibodies specific for the CaV1.2 Ca^2+^-channel subunit (Cat# AGP-001) were used. An anti-glyceraldehyde-3-phosphate dehydrogenase (GAPDH) monoclonal antibody (Santa Cruz Biotechnology, Dallas, TX, USA) was used as an internal loading control.

### General anesthesia

Mice aged 12–16 weeks were anesthetized by placing them in an anesthesia induction chamber (25 × 25 × 14 cm) containing 4% isoflurane (Forane; Abbott Japan Co., Ltd., Tokyo, Japan) and room air. Subsequently, anesthesia was maintained for a 45-min period using 2% isoflurane inhalation anesthesia. For isoflurane inhalation anesthesia, the airflow rate was 0.5 L/min. At 10 min after the induction of anesthesia, basal electrocardiography (ECG) activity was recorded for 5 min, after which pharmacological analyses were performed. All experiments were conducted between 10:00 and 16:00 h.

### ECG evaluation

The ECG activity, heart rate, and R-R interval were measured simultaneously using the ML846 Power Lab system (AD Instruments, Dunedin, New Zealand) [18, 19]. M-button connectors were used to connect the electrodes [18]. Heart rate variability (HRV) is considered an indicator of cardiac autonomic nerve control [18–20]. Our HRV analysis was divided into two separate analyses: time domain analysis based on deviations from the mean R-R interval (standard deviation of normal to normal R-R intervals [SDNN]), and on analysis of the HRV power spectrum. SDNN represents the total variability. The HRV power spectrum consists of three components: very low frequency (VLF), low frequency (LF), and high frequency (HF). Generally, the LF component reflects sympathetic/parasympathetic tone, whereas the HF component reflects parasympathetic tone. The following thresholds were specified for the spectral components according to the manufacturer’s protocol: VLF, < 0.15; LF, < 1.5; and HF, < 5. For pharmacological analyses, mice were administered propranolol (a β-adrenergic blocker, 0.2–0.8 mg/kg) for sympathetic blockade. To observe the baroreflex responses, carotid arteries were ligated for 30 s with 6.0 silk sutures. ECG data were collected using the Power Lab system and analyzed using the LabChart 8 program (ML846 Power Lab system; AD Instruments).

### Echocardiography

Echocardiographic images were obtained using an ultrasound imaging system (Philips Medical Systems, Bothell, WA, USA). Fractional (%) shortening and interventricular septum diameter were evaluated. Mice breathed spontaneously during the echocardiographic studies. Transthoracic M-mode images were obtained from the short axis of the left ventricle (HD11 XE and 15-Hz linear probe; Philips Medical Systems). Left ventricular end-diastole (LVED) and left ventricular end-systole (LVES) were measured. The fractional shortening (%) was calculated using the software supplied with the system.

### Blood pressure measurement

The mouse groups were matched for age and trained several times before the measurements. The heart rate and systolic and diastolic blood pressure were measured using an automated system via the tail-cuff method (BP-98A; Softron, Tokyo, Japan), as described previously [21]. The animals were fed a high-salt diet for 2 weeks before the experiments. Differences were analyzed by repeated-measures analysis of variance.

### Inotropic atrial contraction

The left atria were dissected from the ventricular tissue and placed in an oxygenated tissue bath filled with Tyrode’s solution (123.8 mM NaCl, 5.0 mM KCl, 2.0 mM CaCl_2_, 1.2 mM MgCl_2_, 25.0 mM NaHCO_3_, and 11.2 mM glucose) at 23°C. The isolated left atria were stimulated at 1 Hz with a voltage just above the threshold for basal contraction (1 ms duration). After incubation for 10 min to stabilize the basal contractions, the isometric contractile force was measured using a force transducer (CD200; Nihon Kohden, Tokyo, Japan), as described previously [18]. All data were acquired using the Power Lab system and analyzed using LabChart 8.

### Primary culture of cardiac myocytes

Cardiac myocytes were isolated by Han’s method [22]. Briefly, the heart was quickly excised and cooled in ice-cold cell isolation buffer containing 130 mM NaCl, 5.4 mM KCl, 0.5 mM MgCl_2_, 0.33 mM NaH_2_PO_4_, 22 mM glucose, and 25 mM HEPES (pH 7.4). Isolated cardiac myocytes were cultured in Dulbecco’s modified Eagle’s medium supplemented with 10% fetal bovine serum.

### Monitoring system for PKA activity

To monitor cyclic AMP (cAMP) levels, a PathDetect cis-reporter plasmid, pCRE-hrGFP plasmid, which contains consensus sites for the cAMP response element (four enhancer element repeats of 5′-AGCCTGACGTCAGAG-3′) and a humanized green fluorescent protein (hrGFP) reporter, was purchased from Agilent Technologies (Santa Clara, CA). Isolated cardiac myocytes were plated into 35-mm dishes, and lipofection was performed using commercially prepared regents (Invitrogen). Aliquots of 2 μg DNA were used in each experiment. The cAMP evaluation was performed at least twice for each mouse line.

### Cardiac action potentials

Spontaneously beating right atria were mounted in an organ bath (10 mL) and continuously perfused with oxygenated Tyrode’s solution at 23°C. For cardiac action potential recordings, the hearts were excised and rinsed in normal Tyrode’s solution. The spontaneously beating right atrium was dissected and mounted in the organ bath. Intracellular action potentials were recorded from the intracardiac surface using conventional glass pipettes filled with 3.0 M KCl (tip resistance: 20–30 MΩ).

### Statistical analysis

Results are expressed as mean ± standard error. Means were compared using the Newman-Keuls *post-hoc* multiple-range test. Difference were detected by one-way analysis of variance followed by Dunnett’s *t-*test, and *p*-values < 0.05 were considered significant.

## Results

### Expression profile

**Fig 1:**
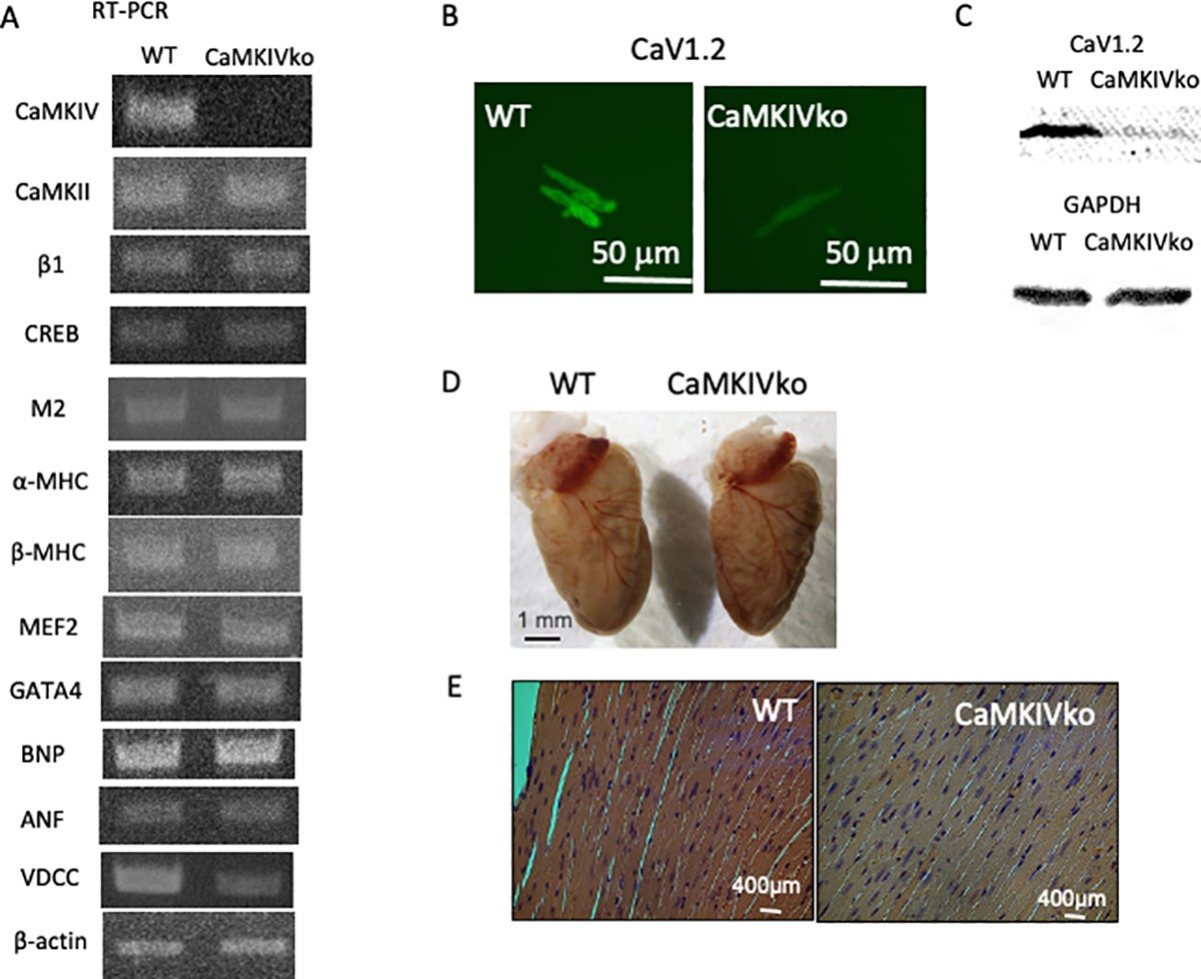
A) Typical RT-PCR data from wild-type (left; WT) and CaMKIV-null (right; CaMKIVko) mice. The amplified sequences are shown. Identification of sympathetic β-adrenergic receptor (β1)-specific transcripts in the heart. CREB1, cholinergic M2 receptor, α-MHC, β-MHC, MEF, GATA4, BNP, ANF, and VDCC transcripts in the heart were also evaluated. Expression of β-actin was evaluated as a control. The primer sets used for PCR amplification are shown. At least four independent RT-PCR outputs were examined for each expression analysis. B) Whole-cell fluorescence immunohistochemistry of CaV1.2 in isolated cardiac myocytes from wild-type and CaMKIV-null mice. Scale bar = 50 μm. CaMKIV-null myocytes showed significantly decreased CaV1.2 levels. C) Western blot analysis of the hearts from wild-type and CaMKIV-null mice. Representative immunoblots of membranes from wild-type and transgenic mice analyzed for the expression of CaV1.2 (α1C) and GAPDH. A significant decrease in the Cav1.2 protein level was observed. n = 6. D) Whole hearts from wild-type (left) and CaMKIV-null (right) mice. E) Histological analysis of hearts from 12-week-old wild-type and CaMKIV-null mice. Hearts were sectioned transversely and stained with hematoxylin and eosin.

To investigate the influence of CaMKIV in the cardiovascular system, we performed RT-PCR analysis of CaMKIV in the heart (Fig 1). Expression of CaMKIV was confirmed in wild-type mice, as reported by Olson’s group [1]. We also confirmed no expression of CaMKIV in the CaMKIV-null mice. The RT-PCR analysis revealed no significant change in CaMKII, the main calcium/calmodulin-dependent protein kinase in the heart. We next explored the expression levels of PKA-related molecules in the heart. No changes in the expression of adrenergic β1 receptor or parasympathetic cholinergic M2 receptor were observed. We also examined CREB1, which is a post-receptor molecule in the adrenergic pathway, and no significant changes were observed.

Expression of cardiac hypertrophy-related genes was further examined. The expression levels of α-MHC, β-MHC, MEF2, transcription factor GATA4, BNP, and atrial natriuretic peptide were apparently unaffected. Interestingly, expression of the VDCC α1 subunit (CaV1.2) was significantly decreased in the CaMKIV-null cardiac myocytes compared with wild-type controls (0.36 ± 0.1 vs. 0.84 ± 0.09 (VDCC/β-actin), respectively, *n* = 6 in each group) (Fig 1A, S1 Fig). β-actin expression was examined as a control.

As RT-PCR analysis revealed a significant decrease in VDCC expression, we examined expression of the CaV1.2 gene by whole-cell fluorescent immunohistochemical analysis in isolated cardiac myocytes (Fig 1B). A limited signal was detected in CaMKIV-null myocytes (53.3 ± 6.7 vs. 28.9 ± 5.6 fluorescent units, respectively, *n* = 16–22) (Fig 1B, S1 Fig). In addition, a western blot analysis with the anti-CaV1.2 antibody confirmed the decreased CaV1.2 protein level in the CaMKIV-null heart (approximately 28% reduction compared to that in the WT heart, *n* = 6, S1 Fig).

### Histology

There is substantial evidence that the intracellular Ca^2+^-binding protein, calmodulin, may be a key regulator of cardiac hypertrophy [16, 23]. Therefore, ablation of the CaMKIV gene may cause cardiac hypotrophy. However, a gross examination under a dissecting microscope revealed no apparent changes in the hearts of 12-week-old CaMKIV-null mice (Fig 1D). We further performed a histological analysis (hematoxylin and eosin staining) of the heart and detected no apparent changes in the hearts of 12-week-old CaMKIV-null mice (Fig 1E). To better understand the cardiomyopathic changes in the heart, the CSA was determined by planimetry (S1 Fig). There were no significant differences between the wild-type and CaMKIV-null mice, indicating that CaMKIV-null mice show marginal hypotrophic changes.

### ECG analysis

**Fig 2:**
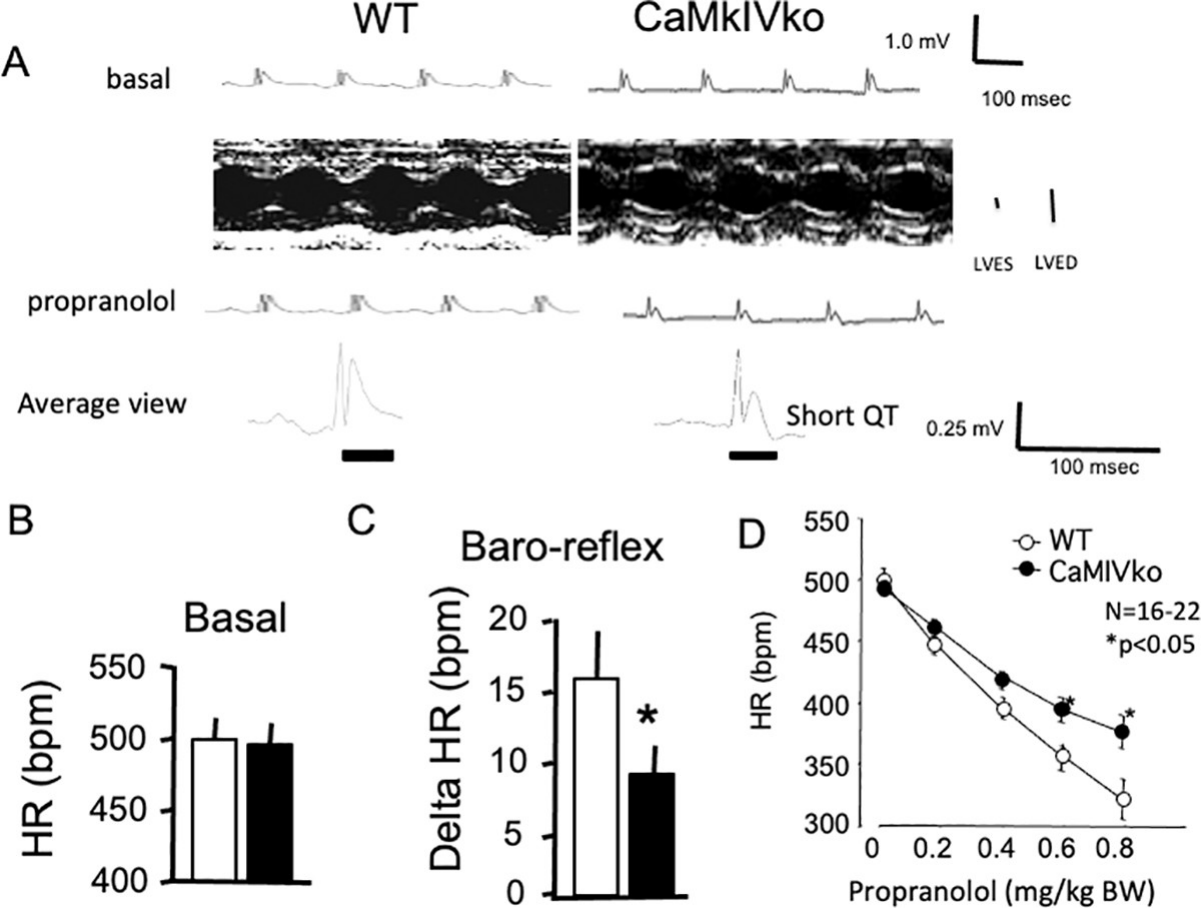
Decreased response to β-blocker (propranolol) in CaMKIV-null mutant mice. A) Representative ECG and echocardiography recordings obtained under basal conditions (upper panels). The left ventricular end-systolic diameter and left ventricular end-diastolic diameter are shown. ECG traces were recorded 10 min after propranolol injection (intraperitoneal injection, lower panels). Scale bar = 100 ms. Representative ECG from wild-type (left panel) and CaMKIV-null (right panel) mice are shown. Note the shortened QT time in CaMKIV-null mice. B) Statistical analysis of the heart rate data of wild-type (*n* = 11; open bars) and CaMKIV-null (*n* = 15; closed bars) mice. There was no significant difference in heart rate between the two groups under basal conditions. C) Decreased baroreflex in CaMKIV-null mice. Statistical analysis of the heart rate changes in wild-type (open bars) and CaMKIV-null (closed bars) mice in response to baroreflex activation. *P < 0.05 between wild-type and CaMKIV-null mice. Each group consisted of six samples. D) Pharmacological manipulation with propranolol. The effects of the β-blocker propranolol (0.2–0.8 mg/kg) are shown. Propranolol administration reduced heart rate changes in CaMKIV-null mice. *P < 0.05 between wild-type and CaMKIV-null mice. Each group consisted of six samples.

As CaMKIV-null heart showed decreased expression of CaV1.2 (α1 subunit of the VDCC), we expected decreased Ca^2+^ influx and cardiac contractility. CaMKIV-null mice had a normal ECG rhythm in the ECG analysis (Fig 2A). Under basal conditions, there were no significant differences in the heart rate between wild-type and CaMKIV-null mice (497.4 ± 16.7 vs. 493.5 ± 9.7 ms, respectively, *n* = 11–13) (Fig 2B). However, the ECG parameter analysis revealed a shortened QT time in CaMKIV-null mice compared with wild-type controls (19.6 ± 5.7 vs. 24.2 ± 2.0 ms, respectively, *N* = 8–15 in each group) (Fig 2A bottom panels). The calculated QT time was also different between the wild-type and CaMKIV-null mice (70.0 ± 6.0 and 57.4 ± 2.5 ms, respectively, N = 8–15). With regard to other ECG parameters, there were no significant differences in PR time or QRS duration between the two groups (S2 Table).

In the echocardiographic recordings, no significant differences were observed in the ejection fraction rate (left ventricular end-systolic diameter/left ventricular end-diastolic diameter) between the wild-type and CaMKIV-null mice (S2 Fig).

The baroreflex showed a decreased response in CaMKIV-null mice compared with wild-type controls (8.9 ± 2.2 vs. 15.6 ± 3.5 ms, respectively, *N* = 6 in each group) (Fig 2C). In the pharmacological analysis, propranolol significantly decreased heart rate in a dose-dependent manner in the wild-type mice (Fig 2D). Propranolol also decreased heart rate in the CaMKIV-null mice, but the responsiveness was decreased at a high dose, indicating a decrease of sympathetic responses in these mice (Fig 2D). No significant difference in the plasma norepinephrine concentration was observed between wild-type and CaMKIV-null mice (1.81 ± 0.27 vs. 1.70 ± 0.23 ng/ml, respectively, *N* = 6 in each group), suggesting that basal sympathetic nerve tone was not modified by the gene manipulation. No significant difference in plasma epinephrine concentration was observed between wild-type and CaMKIV-null mice (1.19 ± 0.22 vs. 1.11 ± 0.20 ng/ml, respectively, *N* = 6 in each group).

We next examined blood pressure as we observed a significant decrease in heart rate changes in response to the non-selective β-blocker propranolol in the ECG analysis of CaMKIV-null mice. No significant differences in basal blood pressure recordings were observed between wild-type and CaMKIV-null mice (S2 Fig).

The nonselective β-agonist isoproterenol was used as a sympathetic nerve stimulus (S2 Fig). Intraperitoneal administration of isoproterenol (0.3 mg/kg) resulted in decreased systolic blood pressure in wild-type mice (−19.5 ± 5.6 mmHg, N = 6), whereas CaMKIV-null mice exhibited a significantly decreased response (−6.7 ± 1.3 mmHg, N = 6, P < 0.05). Isoproterenol also increased heart rate in wild-type mice (30.7 ± 8.1 bpm, N = 6), whereas it induced only a slight increase in CaMKIV-null mice (7.8 ± 6.2 bpm, N = 6, P < 0.05), indicating that the CaMKIV-null mice exhibited decreased responsiveness to β-agonists. However, we found no significant difference in the response to the α1-agonist phenylephrine between wild-type and CaMKIV-null mice (S2 Fig).

Collectively, the present results suggest decreased responsiveness to β-adrenergic stimulation in CaMKIV-null mice.

### HRV changes

**Fig 3:**
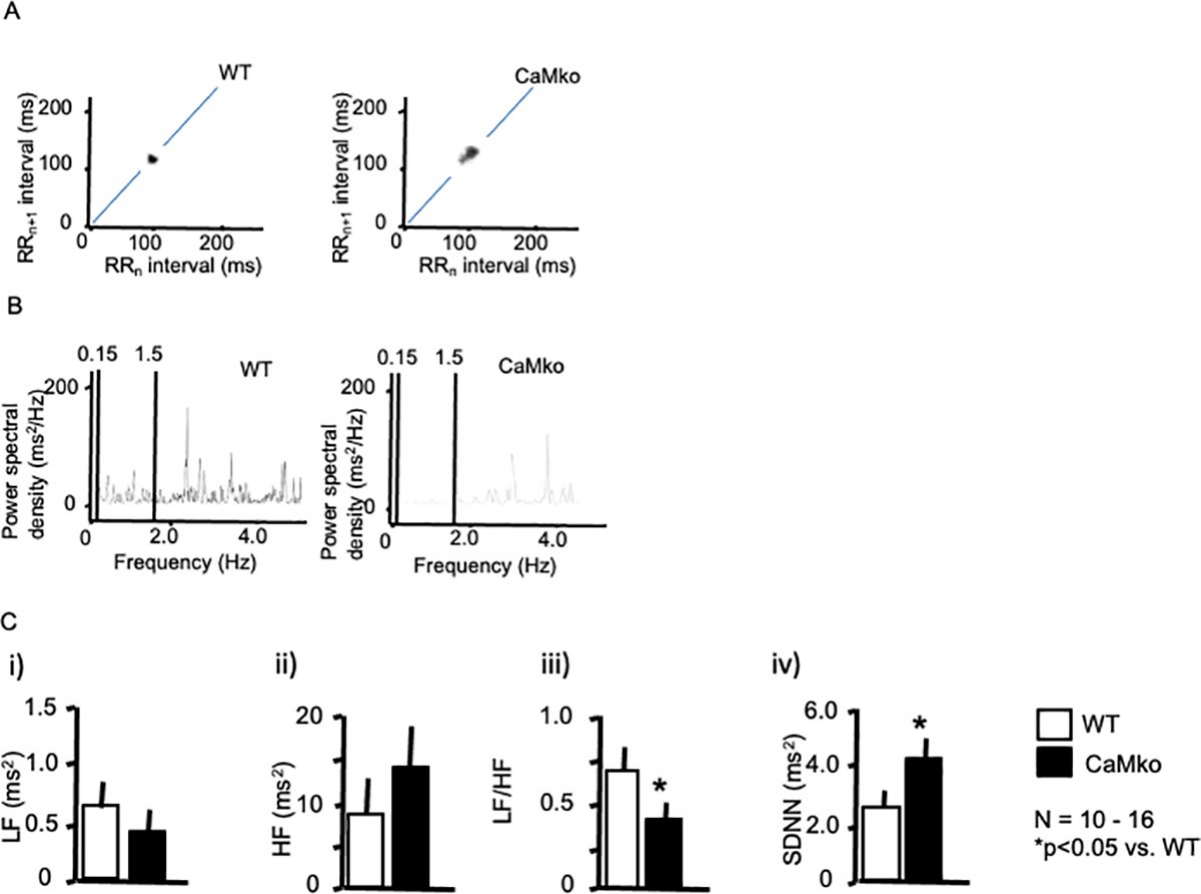
HRV analysis. A) Representative ECG Poincaré plots of wild-type (left panel) and CaMKIV-null mice (right panel). Poincaré plots (RRn vs. RRn+1), in which consecutive pairs of R-R intervals during the control period were plotted, along with the nth+1 R-R interval, against the nth R-R period. Note the marked scattering of CaMKIV-null mice. B) Representative power spectral analysis of wild-type (left panel) and CaMKIV-null mice (right panel). C) Statistical analysis of the power spectra (i–iii). The LF (i) and HF (ii) components, LF/HF ratio (iii), and SDNN (iv) of wild-type (open bars) and CaMKIV-null mice (closed bars). *P < 0.05 vs. wild-type. Each group consisted of at least 10 samples. Error bars indicate the SEM.

CaMKIV-null mice exhibited decreased responsiveness to the β-adrenergic blocker propranolol. Therefore, we performed an R-R interval calculation to analyze HRV. HRV is among the methods used to evaluate cardiac autonomic nerve control [24]. We performed ECG analysis using Power Lab HRV software to explore heart rate regulation. Fig 3A shows representative Poincaré plots for wild-type (left panel) and CaMKIV-null mice (right panel). Wild-type mice exhibited stable R-R intervals, whereas CaMKIV-null mice had relatively unstable R-R intervals (shown as dots), indicating unstable pacemaking.

In the frequency domain analysis, LF (0.2–0.75 Hz) and HF (0.75–2.5 Hz) components were resolved in the power spectral density (Fig 3B). Power spectral analysis (Fig 3B and 3C) showed that the LF/HF ratio was decreased in the CaMKIV mutants (0.34 ± 0.07, N = 15, P < 0.05) compared with wild-type mice (0.70 ± 0.09, N = 10), although no statistically significant differences were observed in the LF or HF components (Fig 3C), suggesting a limited contribution of CaMKIV to the sympathetic signal transduction system. Nevertheless, the significant decrease in the LF/HF in CaMKIV-mutant mice also supports the idea of decreased sympathetic responsiveness in these animals. Regarding the SDNN calculation, CaMKIV-null mice showed a significantly increased SDNN compared with wild-type controls (4.17 ± 0.85 vs. 2.65 ± 0.75 ms^2^, respectively, N = 10–16 in each group) (Fig 3Civ), in accordance with Poincaré plots.

Taken together, the decreased baroreflex and responsiveness to propranolol in the CaMKIV-null mice revealed an important role of CaMKIV kinase in sympathetic signal transduction.

### Inotropic atrial contraction

**Fig 4:**
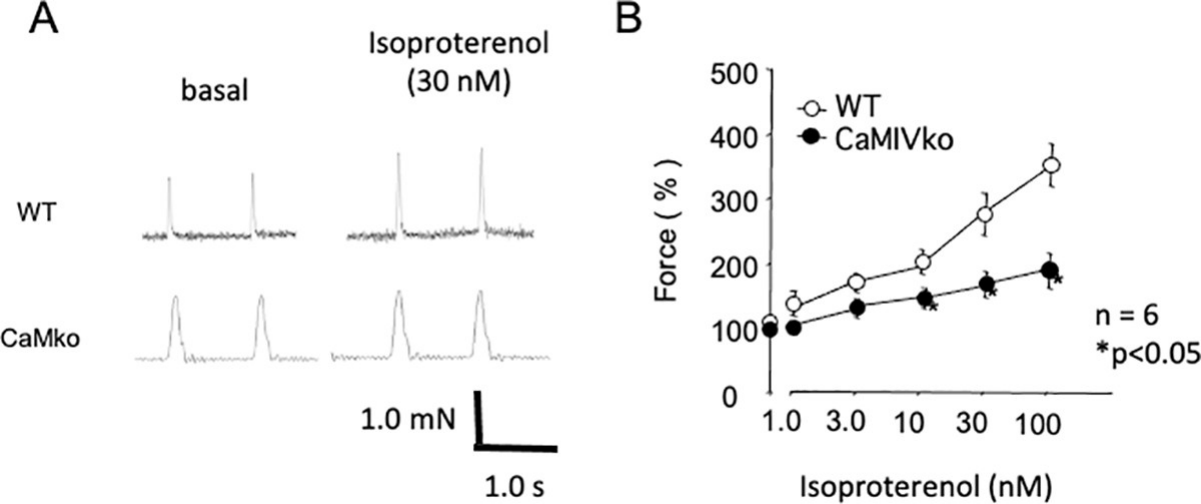
Decreased contractility in CaMKIV-null atria in response to isoproterenol. A) Typical traces of atrial contraction in wild-type (WT, upper panels) and CaMKIV-null (CaMKIVko, lower panels) atria. Isoproterenol (30 nM) increased contractility in the wild-type and CAMKIV-null atria. B) Dose-dependent changes in contractility in response to isoproterenol (1.0–100 nM) in wild-type (open circles) and CaMKIV-null (closed circles) atria. Error bars indicate SEM. *P < 0.05 between wild-type and CaMKIV-null atria.

As modified sympathetic signal transduction in CaMKIV-null cardiac myocytes may decrease responsiveness, as reflected in the heart rate changes in the ECG recordings, we assessed sympathetic responsiveness by measuring inotropic responses to electrical field stimulation in isolated left atria. Fig 4 shows the dose-dependent changes in response to isoproterenol (1–100 nM) in the wild-type (upper panel) and CaMKIV-null (lower panel) atria. Isoproterenol caused dose-dependent changes in force in the atria of wild-type and CaMKIV-null mice, whereas CaMKIV-null atria showed decreased responsiveness at high doses (Fig 4B).

As a decreased response to isoproterenol was observed in the CaMKIV-null atrium, we evaluated changes in the ejection fraction in response to isoproterenol (4 μg/kg) by echocardiogram. CaMKIV-null mice exhibited decreased responsiveness to isoproterenol (S2 Fig).

### Action potential recordings in the atrium

**Fig 5:**
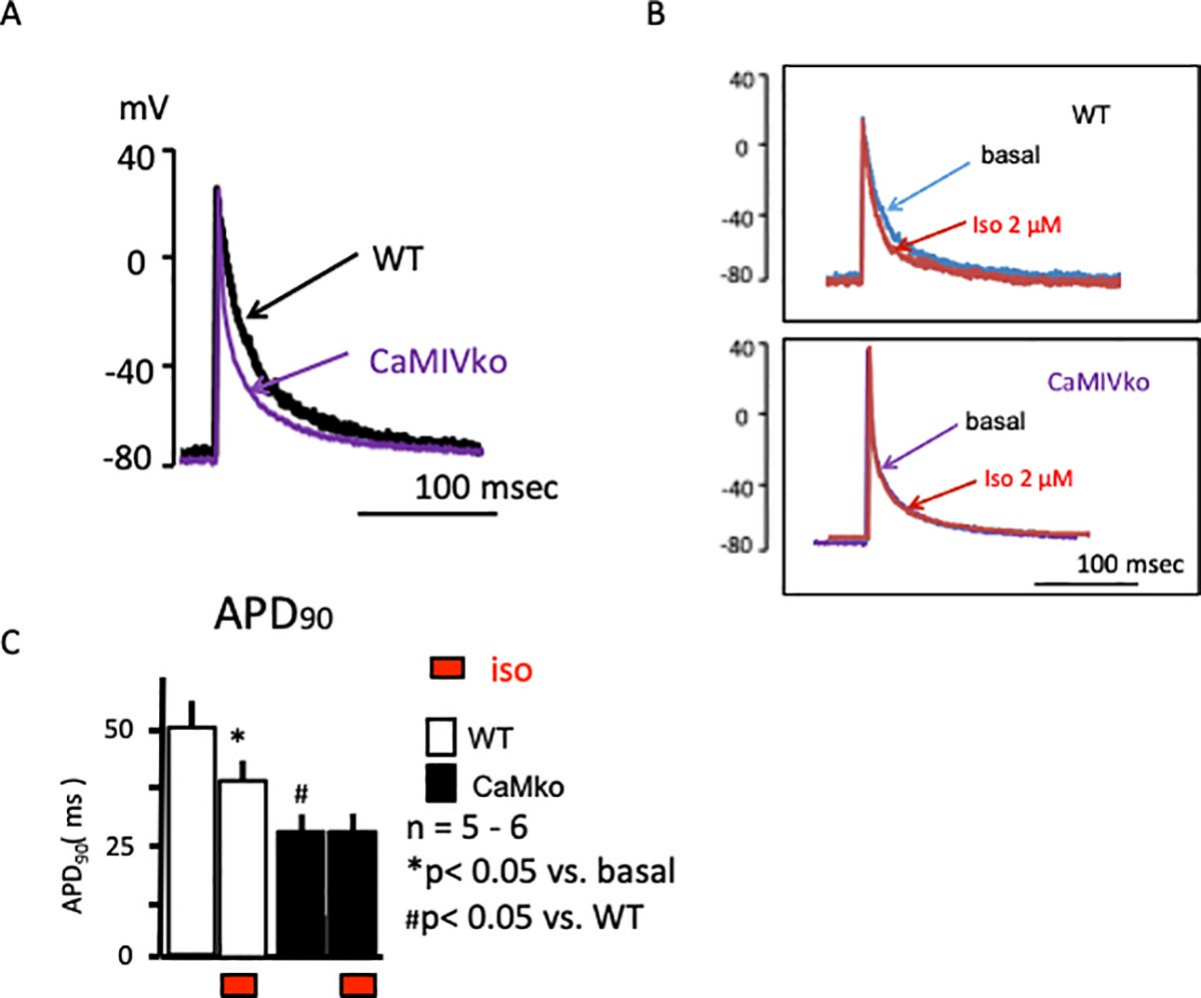
A) Representative cardiac action potential recordings of wild-type (WT, black) and CaMKIV-null (CaMKIVko, purple) atria under basal conditions. B) Representative cardiac action potential recordings in the atrium and response to isoproterenol (red) in wild-type (upper panel) and CaMKIV-null (lower panel) atria. C) Statistical analyses revealed a significant reduction in action potential duration at 90% (APD_90_) in response to isoproterenol in wild-type atria (open bar). CaMKIV-null atria showed a shortened APD_90_ under basal conditions (closed bar). Isoproterenol had a marginal effect on APD_90_ in CaMKIV-null atria.

To further evaluate the electrophysiological properties of the hearts of mice with CaMKIV gene ablation, we evaluated membrane action potentials in the atrium (Fig 5). The wild-type atrium showed rapid increases in the cardiac action potential, followed by a plateau phase (black line in Fig 5A). Interestingly, the CaMKIV-null atrium showed shorter action potentials (purple line in Fig 5A) in comparison with the wild-type atrium. Isoproterenol (2 μM) shortened the action potentials in the wild-type atrium (red line in Fig 5B, upper panel). On the other hand, the CaMKIV-null atrium showed a marginal response to isoproterenol (red line in Fig 5B, lower panel). Atrial action potential duration at 90% repolarization (APD_90_) was significantly shorter in the CaMKIV-null than wild-type atrium under basal conditions (Fig 5C). Isoproterenol shortened the action potentials in the wild-type mice, while CaMKIV-null mice showed no significant changes in response to isoproterenol. Taken together, the results of the action potential analysis revealed decreased sympathetic responsiveness in CaMKIV-null mice, consistent with the ECG and atrial contraction analyses.

### Fluorescence imaging of cAMP

**Fig 6:**
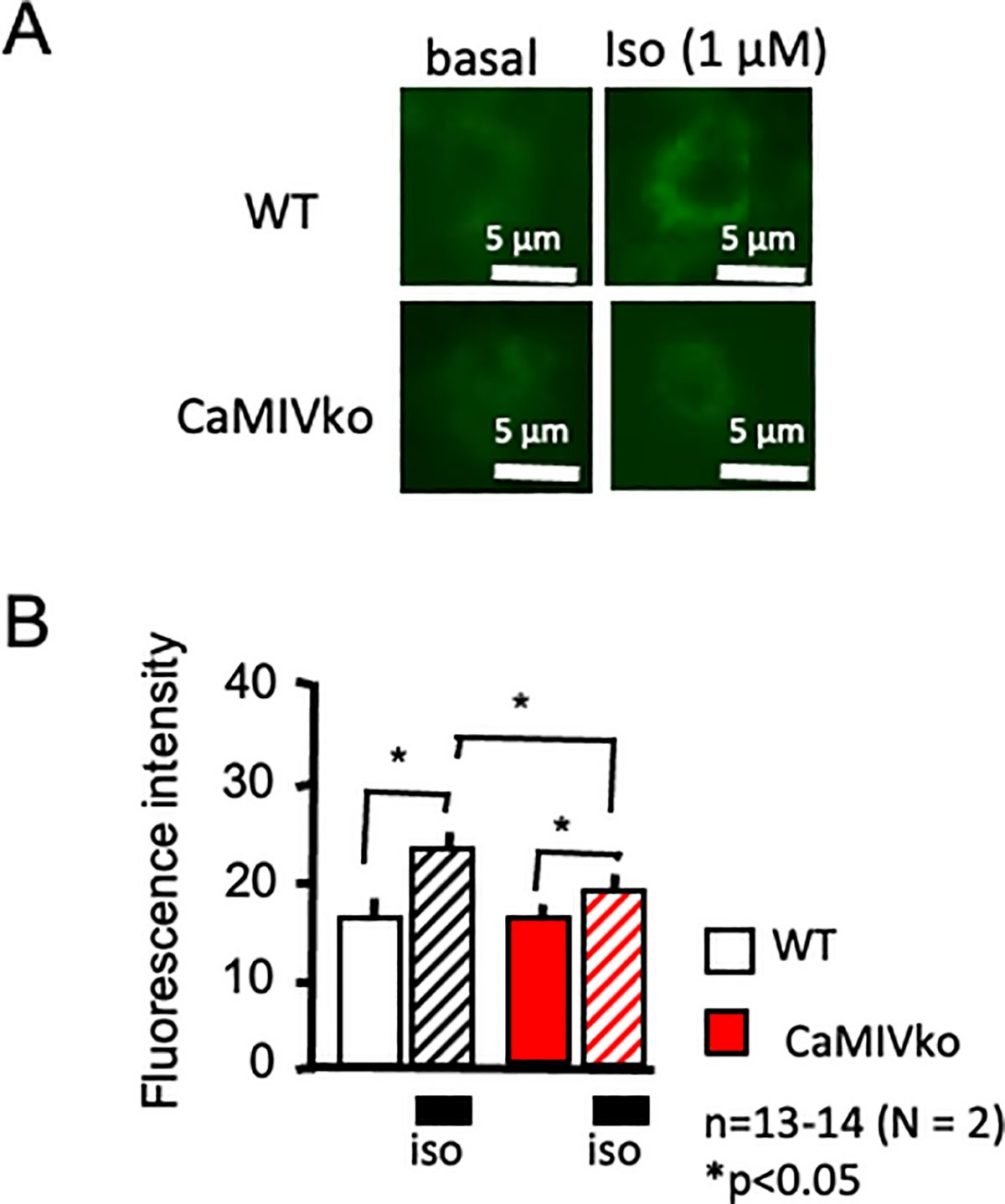
Expression analysis using a CREB-EGFP assay. A) Representative CREB-EGFP fluorescence. In wild-type cardiac myocytes, isoproterenol showed increased fluorescence, indicating increased cAMP levels (upper panels, wild-type [WT]). CaMKIV-null (CaMKIVko) myocytes showed a limited response (lower panels). B) Statistical analysis revealed increased CREB-EGFP fluorescence in the wild-type myocytes (open and hatched bars), whereas limited changes were observed in CaMKIV-null myocytes (red and red-hatched bars). CREB-EGFP activity was assessed according to the level of GFP fluorescence at 48 h after transfection (*n* = 13–14). The results are presented as the means ± SEM. *P < 0.05. Scale bar = 5 μm.

CaMKIV-null mice exhibited decreased responsiveness to a β-adrenergic stimulus, so we next analyzed intracellular changes in the cAMP level. A CRE-hrGFP assay was performed at 48 h after transfection. The intracellular cAMP level was evaluated based on the EGFP fluorescence intensity. As shown in Fig 6A, isoproterenol (1 μM) stimulation significantly increased EGFP fluorescence in the wild-type myocytes (upper panels). On the other hand, a relatively small enhancement was detected in the CaMKIV-null myocytes (lower panels). CaMKIV-null myocytes showed significantly decreased responsiveness to isoproterenol in comparison with wild-type controls (Fig 6B).

## Discussion

In the present study, we examined the importance of CaMKIV kinase in cardiac function. CaMKIV-null mice showed limited changes in heart rate in response to baroreflex in comparison with wild-type mice. Interestingly, expression of voltage-dependent Ca^2+^ channels was significantly decreased in CaMKIV-null hearts. CaMKIV-null mice showed a short QT time, which may be due to decreased Ca^2+^ currents. Decreased responses to propranolol, a β-adrenergic blocker, and isoproterenol, a β-adrenoceptor agonist, indicated that CaMKIV-null mice have attenuated β-adrenergic responsiveness.

Our physiological analysis revealed decreased sympathetic response in CaMKIV-null heart. Under basal conditions, we found no significant differences in heart rate or ejection fraction between wild-type and CaMKIV-null mice, but CaMKIV-null mice showed attenuated responses to adrenergic modulation (β-blocker or β-agonist). These findings suggest that CaMKIV plays an important role(s) in sympathetic nerve signal transduction in cardiac myocytes.

In the HRV analysis, CaMKIV-null mice showed increased SDNN. As basal heart rate was not different between CaMKIV-null and wild-type mice, it is obvious that CaMKIV-null mice have a more unstable R-R interval than wild-type mice, suggesting decreased autonomic nerve regulation, similar to the voltage-dependent β3 subunit of the calcium channel β3 in null-mutant mice, which have a variable R-R interval because there are fewer N-type calcium channels [24, 25]. The sympathetic and parasympathetic nervous systems likely have effects on LF and HF in a nonlinear manner [26]. Particularly, the relationship between LF power and cardiac sympathetic regulation is affected by cholinergic antagonists [26]. The calculated HRV parameters produce complex LF, HF, and LF/HF data. We speculate that the LF/HF data may not be appropriate to accurately quantify cardiac “sympathovagal balance” [26]. One advantage of an HRV analysis is that it assesses the effects of various manipulations using simple ECG recordings. Although we observed decreased responsiveness to β-adrenergic stimulation in the isolated atrium, which indicated attenuation of β-adrenergic response in calcium/calmodulin-dependent protein kinase IV-knockout mice, additional analysis is needed on the pharmacological effects of the α-adrenergic receptor agonist phenylephrine, and sodium nitroprusside, on peripheral vascular resistance.

A number of studies indicated that alterations in intracellular Ca^2+^ signaling are primary stimuli for a hypertrophic response [10–12]. Passier et al. generated a transgenic mouse line with overexpression of constitutively active CaMKIV [1] and reported that overexpression of this gene resulted in cardiac hypertrophy and activation of the MEF2 transcription factor *in vivo*. Overexpression of constitutively active CaMKIV resulted in increased arrhythmia, which was probably related to elongated QT time.

Intracellular calcium is important for calcium/calmodulin kinase, which is calcium dependent. CaM kinase phosphorylates CREB and affects the transcription of various genes. This decreased expression of CaV1.2 indicates that this cascade may be involved, although further analysis is needed.

The RT-PCR analysis revealed marginal effects on transcription factors of CaMKIV gene ablation. However, detection sensitivity may be limited with conventional RT-PCR, as used in the present study. Nevertheless, no significant changes in the expression of MEF2 in CaMKIV-null mice were observed in the present study. Ablation of the CaMKIV gene also showed no hypotrophic changes in cardiac myocytes. Considering the unchanged expression of CaMKII in the CaMKIV-null heart, the effect on gene expression may be limited. An expression analysis may reveal how potassium channel genes form IK1, which reflects how the cardiac action potential and other potassium channels including IKs or IKr could be affected by CaMKIV gene manipulation [27]. PKA-dependent channel phosphorylation of the cardiac ryanodine receptor type-2 (RyR-2) is also known. PKA phosphorylation of phospholamban is important for rapid Ca^2+^ uptake into sarcoplasmic reticulum, resulting in positive chronotropic action of β-adrenergic stimulation. Nevertheless, further studies of cardiac PKA-related molecules in CaMKIV-null mice are needed.

In the present study, CaMKIV-null mice showed a short QT time. Q-waves in ECG represent the phase of ventricular depolarization, which is due mainly to the plateau phase of cardiac action potential voltage. As the plateau phase of the cardiac action potential relies mainly on Ca^2+^ influx via L-type voltage-dependent Ca^2+^ channels, decreased expression of CaV1.2 in CaMKIV-null mice (Fig 1) may be related to the shortened Q-waves.

Interestingly, cardiac action potential recording indicated a short APD duration and decreased APD_90_ changes in response to isoproterenol. These findings appear to be related to decreased PKA-related phosphorylation. As discussed above, depolarization of the cardiac action potential is related to Ca^2+^ influx via voltage-dependent Ca^2+^ channels. Phosphorylation of the Ca^2+^ channels by PKA is one of the main phenomena involved in increasing cardiac contractile force during sympathetic nerve activation [28]. Therefore, we evaluated PKA-dependent phosphorylation using a CREB-EGFP construct. In wild-type cardiac myocytes, isoproterenol increased CREB-related EGFP fluorescence, whereas CaMKIV-null cardiac myocytes showed limited changes. These data strongly suggest that CaMKIV-null mice have decreased PKA-dependent phosphorylation. Taken together, the results of the present study strongly suggest that CaMKIV is somehow related to PKA-dependent phosphorylation.

In conclusion, CaMKIV-null mice showed attenuated sympathetic nerve responses. CaMKIV-null mice showed decreased CaV1.2 expression in cardiac myocytes, short APD_90_, and short QT time duration, indicating decreased Ca^2+^ influx via voltage-dependent Ca^2+^ channels. The results of the present study suggest that CaMKIV plays an important role(s) in sympathetic nerve signal transduction in cardiac myocytes.

## Supporting information

S1 Fig A. RT-PCR of VDCC in wild-type (open bars) and CaMKIV1-null (closed bars) hearts. *P < 0.05 between wild-type and CaMKIV-null mice. Each group consisted of six samples. B. Fluorescent immunostaining of VDCC in wild-type (open bar) and CaMKIV-null (closed bar) cardiac myocytes. *P < 0.05, between wild-type and CaMKIV-null mice. Each group consisted of 16–22 cells. C. Statistical analysis of immunoblotting for CaV1.2 and GAPDH. *P < 0.05 between wild-type and CaMKIV-null mice. Each experiment was repeated six times. All values are mean ± standard error. D. Statistical analysis of cross-sectional areas of wild-type (open bars) and CaMKIV-null (closed bars) hearts. Each group consisted of six samples.
(PDF)

S2 Fig A. Basal systolic blood pressure. Basal systolic blood pressure (SBP) in wild-type (WT) (open bar) and CaMKIV-null (solid bar). *N* = 6. B. Response to isoproterenol. The SBP and heart rate (HR) after administration of isoproterenol (0.3 mg/kg). CaMKIV-null mice showed significantly decreased blood pressure and HR changes in response to isoproterenol. *P < 0.05 between wild-type and CaMKIV-null mice. Each group consisted of at least six samples. C. Pharmacological response to phenylephrine. Systolic blood pressure (SBP) and heart rate (HR) after administering phenylephrine (30 μg/kg). Each group consisted of at least six samples. Echocardiographic analysis of wild-type (open bars) and CaMKIV-null (closed bars) mice. D. Basal ejection fraction. Under basal conditions, no significant differences were observed in the ejection fraction. E. Changes in the ejection fraction in response to isoproterenol. Isoproterenol increased the ejection fraction in wild-type mice, whereas CaMKIV-null mice showed limited changes in response to isoproterenol. *P < 0.05, between wild-type and CaMKIV-null mice. Each group consisted of at least six samples.
(PDF)

S3 Fig A. Original RT-PCR gels. Primer sets are indicated. B. Original western blots. The proteins examined are indicated.
(PDF)

S1 File (DOCX)

S1 Table Oligo DNAs used in RT-PCR analyses. (PDF)

S2 Table ECG parameters in Wild-Type (WT) and CaMKIV1-null (CaMKIVko) mice. Each group consisted of at least 10 mice.
(PDF)
