# Supplementary material for: Attenuated β-adrenergic response in calcium/calmodulin-dependent protein kinase IV-knockout mice
Source: PLoS One. 2021 Apr 15;16(4):e0249932. doi: 10.1371/journal.pone.0249932 (PMC8049319; doi:10.1371/journal.pone.0249932)
Supplement: S1 Fig — A. RT-PCR of VDCC in wild-type (open bars) and CaMKIV1-null (closed bars) hearts. *P < 0.05 between wild-type and CaMKIV-null mice. Each group consisted of six samples. B. Fluorescent immunostaining of VDCC in wild-type (open bar) and CaMKIV-null (closed bar) cardiac myocytes. *P < 0.05, between wild-type and CaMKIV-null mice. Each group consisted of 16–22 cells. C. Statistical analysis of immunoblotting for CaV1.2 and GAPDH. *P < 0.05 between wild-type and CaMKIV-null mice. Each experiment was repeated six times. All values are mean ± standard error. D. Statistical analysis of cross-sectional areas of wild-type (open bars) and CaMKIV-null (closed bars) hearts. Each group consisted of six samples. (PDF) [file pone.0249932.s001.pdf]

S1 Fig.

A. RT-PCR of VDCC in wild-type (open bars) and CaMKIV1-null (closed bars) hearts. $^*P < 0.05$ between wild-type and CaMKIV-null mice. Each group consisted of six samples.

B. Fluorescent immunostaining of VDCC in wild-type (open bar) and CaMKIV-null (closed bar) cardiac myocytes. $^*P < 0.05$, between wild-type and CaMKIV-null mice. Each group consisted of 16–22 cells.

C. Statistical analysis of immunoblotting for CaV1.2 and GAPDH. $^*P < 0.05$ between wild-type and CaMKIV-null mice. Each experiment was repeated six times. All values are mean ± standard error.

D. Statistical analysis of cross-sectional areas of wild-type (open bars) and CaMKIV-null (closed bars) hearts. Each group consisted of six samples.

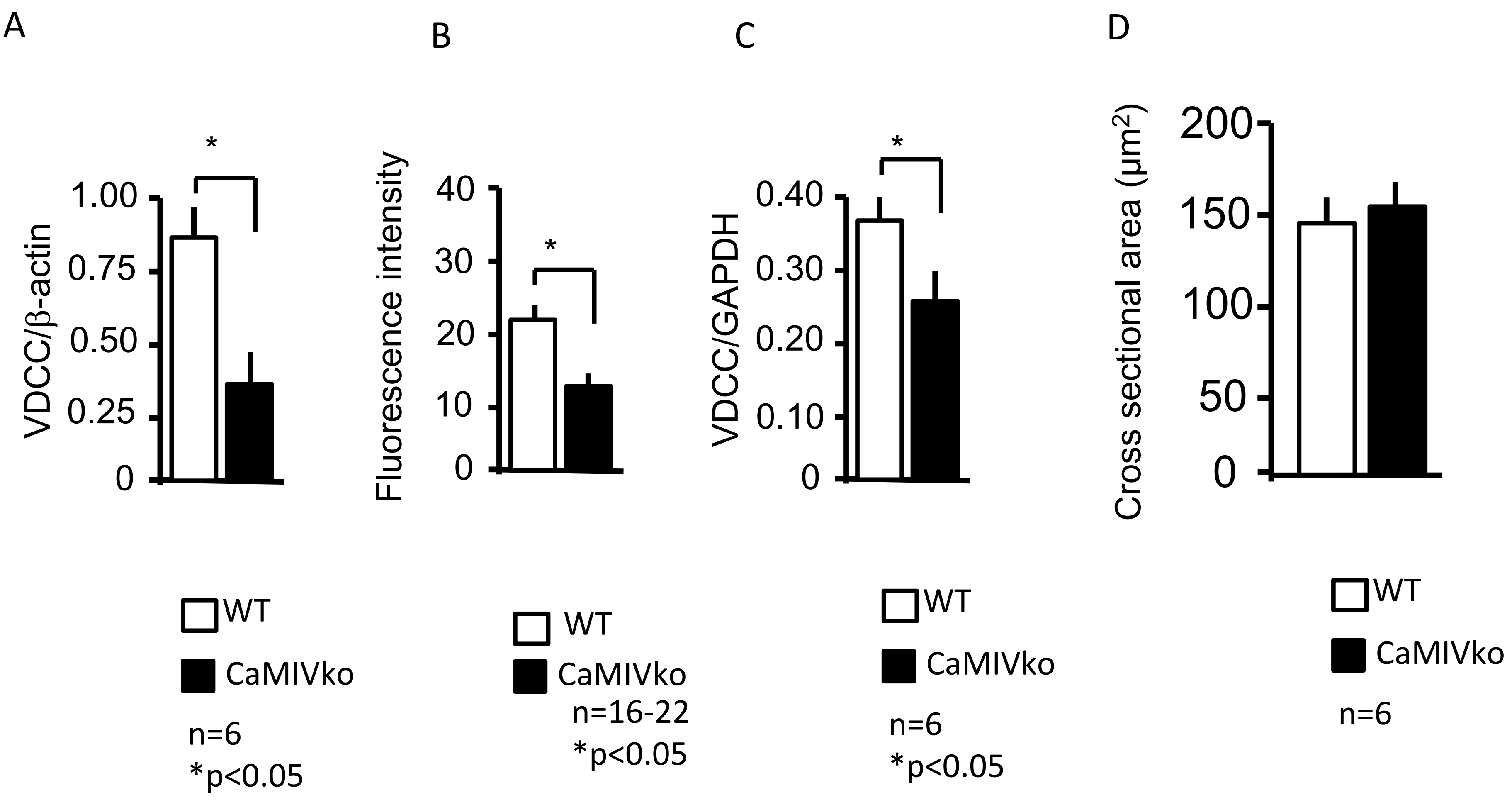

A
B
C
D
*
VDCC/β-actin
1.00
0.75
0.50
0.25
0
Fluorescence intensity
40
30
20
10
0
VDCC/GAPDH
0.40
0.30
0.20
0.10
0
Cross sectional area (μm2)
200
150
100
50
0
WT
CaMIVko
n=6
*p<0.05
n=16-22
*p<0.05
n=6
*p<0.05
n=6
