# Supplementary material for: Attenuated β-adrenergic response in calcium/calmodulin-dependent protein kinase IV-knockout mice
Source: PLoS One. 2021 Apr 15;16(4):e0249932. doi: 10.1371/journal.pone.0249932 (PMC8049319; doi:10.1371/journal.pone.0249932)
Supplement: S2 Fig — A. Basal systolic blood pressure. Basal systolic blood pressure (SBP) in wild-type (WT) (open bar) and CaMKIV-null (solid bar). N = 6. B. Response to isoproterenol. The SBP and heart rate (HR) after administration of isoproterenol (0.3 mg/kg). CaMKIV-null mice showed significantly decreased blood pressure and HR changes in response to isoproterenol. *P < 0.05 between wild-type and CaMKIV-null mice. Each group consisted of at least six samples. C. Pharmacological response to phenylephrine. Systolic blood pressure (SBP) and heart rate (HR) after administering phenylephrine (30 μg/kg). Each group consisted of at least six samples. Echocardiographic analysis of wild-type (open bars) and CaMKIV-null (closed bars) mice. D. Basal ejection fraction. Under basal conditions, no significant differences were observed in the ejection fraction. E. Changes in the ejection fraction in response to isoproterenol. Isoproterenol increased the ejection fraction in wild-type mice, whereas CaMKIV-null mice showed limited changes in response to isoproterenol. *P < 0.05, between wild-type and CaMKIV-null mice. Each group consisted of at least six samples. (PDF) [file pone.0249932.s002.pdf]

S2 Fig.

A. Basal systolic blood pressure

Basal systolic blood pressure (SBP) in wild-type (WT) (open bar) and CaMKIV-null (solid bar). $N = 6$.

B. Response to isoproterenol

The SBP and heart rate (HR) after administration of isoproterenol (0.3 mg/kg). CaMKIV-null mice showed significantly decreased blood pressure and HR changes in response to isoproterenol. $^{*}P < 0.05$ between wild-type and CaMKIV-null mice. Each group consisted of at least six samples.

C. Pharmacological response to phenylephrine

Systolic blood pressure (SBP) and heart rate (HR) after administering phenylephrine (30 μg/kg). Each group consisted of at least six samples.

Echocardiographic analysis of wild-type (open bars) and CaMKIV-null (closed bars) mice.

D. Basal ejection fraction

Under basal conditions, no significant differences were observed in the ejection fraction.

E. Changes in the ejection fraction in response to isoproterenol

Isoproterenol increased the ejection fraction in wild-type mice, whereas CaMKIV-null mice showed limited changes in response to isoproterenol. $^{*}P < 0.05$, between wild-type and CaMKIV-null mice. Each group consisted of at least six samples.

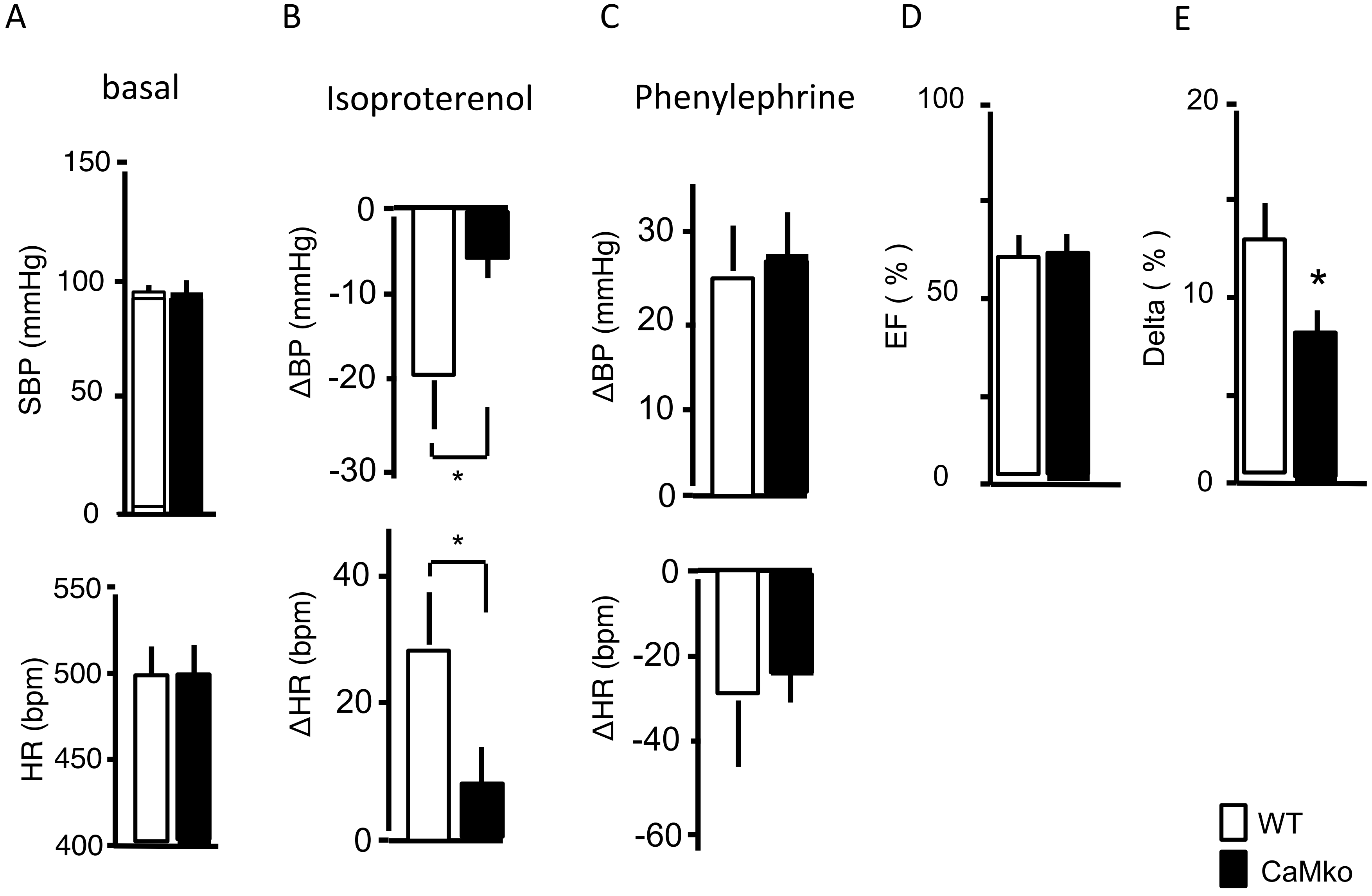
A
B
C
D
E
basal
Isoproterenol
Phenylephrine
SBP (mmHg)
150
100
50
0
HR (bpm)
550
500
450
400
ΔBP (mmHg)
0
-10
-20
-30
*
ΔHR (bpm)
40
20
0
*
ΔBP (mmHg)
30
20
10
0
ΔHR (bpm)
0
-20
-40
-60
EF (%)
100
50
0
Delta (%)
20
10
0
*
WT
CaMko
