# Supplementary material for: Attenuated β-adrenergic response in calcium/calmodulin-dependent protein kinase IV-knockout mice
Source: PLoS One. 2021 Apr 15;16(4):e0249932. doi: 10.1371/journal.pone.0249932 (PMC8049319; doi:10.1371/journal.pone.0249932)
Supplement: S3 Fig — A. Original RT-PCR gels. Primer sets are indicated. B. Original western blots. The proteins examined are indicated. (PDF) [file pone.0249932.s003.pdf]

S3 Fig.

A. Original RT-PCR gels

Primer sets are indicated.

B. Original western blots

The proteins examined are indicated.

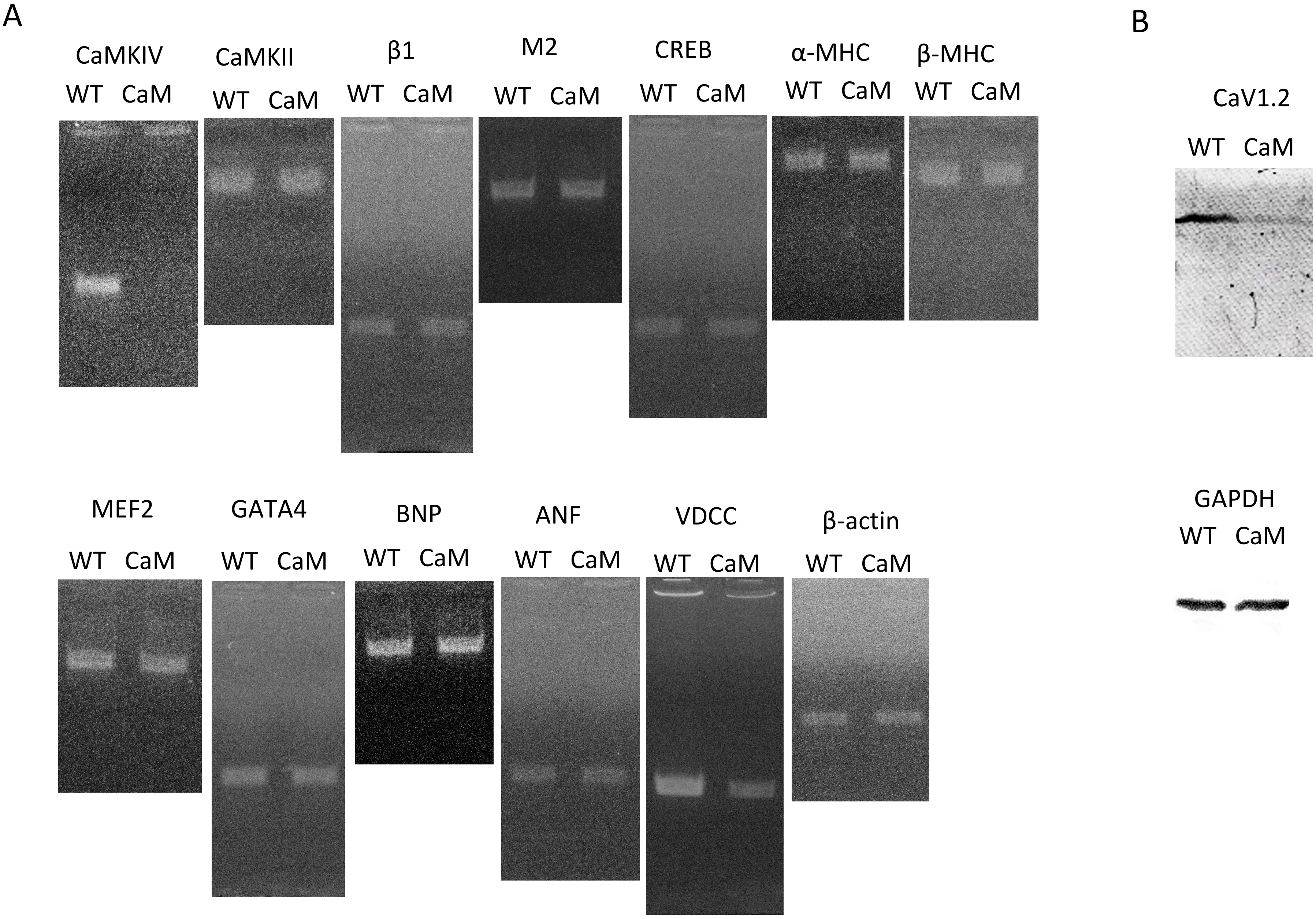
A
CaMKIV
WT CaM
CaMKII
WT CaM
β1
WT CaM
M2
WT CaM
CREB
WT CaM
α-MHC
WT CaM
β-MHC
WT CaM
MEF2
WT CaM
GATA4
WT CaM
BNP
WT CaM
ANF
WT CaM
VDCC
WT CaM
β-actin
WT CaM
B
CaV1.2
WT CaM
GAPDH
WT CaM
