# Supplementary material for: Attenuated β-adrenergic response in calcium/calmodulin-dependent protein kinase IV-knockout mice
Source: PLoS One. 2021 Apr 15;16(4):e0249932. doi: 10.1371/journal.pone.0249932 (PMC8049319; doi:10.1371/journal.pone.0249932)
Supplement: S1 File — (DOCX) [file pone.0249932.s004.docx]

**Limitations**

In this study, we calculated HRV to evaluate sympathetic modulation due to CaMKIV ablation. ECG and HRV analyses are relatively simple compared with electrophysiological patch-clamp analysis, which directly records the excitability of neurons. However, accurate HRV calculations and evaluation of autonomic nerve status are difficult, as they are affected by other factors, such as respiration.

Marginal effects on transcription factors of CaMKIV gene ablation were revealed by the RT-PCR analysis. We suspect that detection sensitivity may be limited by conventional RT-PCR, which was used in the present study. Quantitative PCR (qPCR) and real-time PCR, which incorporates fluorescent dyes during PCR amplification, are widely used in clinical research.

Electrophysiological analysis using the patch-clamp technique should be applied to CaMKIV-null cardiac myocytes, although such evaluations of single cells are relatively complex and time-consuming.
