# Supplementary material for: Attenuated β-adrenergic response in calcium/calmodulin-dependent protein kinase IV-knockout mice
Source: PLoS One. 2021 Apr 15;16(4):e0249932. doi: 10.1371/journal.pone.0249932 (PMC8049319; doi:10.1371/journal.pone.0249932)
Supplement: S1 Table — (PDF) [file pone.0249932.s005.pdf]

S1 Table

Oligo DNAs used in RT-PCR analyses

	Forward	Reverse
CaMKIV	CGATTTCTTCGAGGTGGAGT	CCCCTTCTGTTTGCATCTGT
CaMII	GCTTTCAGCCAGAGATCACC	CAGTGTAGCACAGCCTCCAA
β1	CATCGTTCTGCTCATCGTGG	ACACACAGCACATCTACCGA
CREB	CCAAACTAGCAGTGGGCAGT	TGTACATCGCCTGAGGCAGC
M2	GGTAAGGACTGTGGAAGA	ACCAGGCATGTTGTTGTTG
α-MHC	CGACCTCAACGAGATGGAGA	TGGCGATGTTCTCCTTCAGG
β-MHC	TGCAGCAGTTCTTCAACCAC	TCGAGGCTTCTGGAAGTTGT
MEF2	TGATCAGCAGGCAAAGATTG	ATCAGACCGCCTGTGTTACC
GATA4	GCAGCAGCAGTGAAGAGATG	GCGATGTCTGAGTGACAGGA
BNP	AGTCCTAGCCAGTCTCCAGA	ATCCGGTCTATCTTGTGCCC
ANF	GCCGGTAGAAGATGAGGTCA	AGCTGGATCTTCGTAGGCTC
VDCC	CCCTTCTTGTGCTCTTCGTCATCA	GGCTTTGGCTTTCTCCCTCTCT
β-actin	CAACTGGGACGACATGGAGAA	CAGCCTGGATGGCTACGTACA
