# Supplementary material for: Attenuated β-adrenergic response in calcium/calmodulin-dependent protein kinase IV-knockout mice
Source: PLoS One. 2021 Apr 15;16(4):e0249932. doi: 10.1371/journal.pone.0249932 (PMC8049319; doi:10.1371/journal.pone.0249932)
Supplement: S2 Table — Each group consisted of at least 10 mice. (PDF) [file pone.0249932.s006.pdf]

S2 Table

ECG parameters in wild-type (WT) and CaMKIV1-null (CaMKIVko) mice.

Each group consisted of at least 10 mice.

	RR Interval (msec)	PR Interval (msec)	QRS Interval (msec)
WT	123.5 ± 4.3	37.6 ± 1.7	10.0 ± 0. 4
CaMKIVko	118.0 ± 3.1	35.4 ± 1.2	10.7 ± 0. 5

N = 10 - 14
